# Keeping the horse with the cart: are we underdosing tazobactam even when using continuous-infusion ceftolozane/tazobactam for effectively preventing resistance development by ESBL-producing Enterobacterales?

**DOI:** 10.1128/aac.01215-25

**Published:** 2025-10-29

**Authors:** Manjunath P. Pai, Pier Giorgio Cojutti, Milo Gatti, Matteo Rinaldi, Tommaso Tonetti, Antonio Siniscalchi, Pierluigi Viale, Federico Pea

**Affiliations:** 1 Department of Clinical Pharmacy, College of Pharmacy, University of Michigan 15514, Ann Arbor, Michigan, USA; 2 Department of Medical and Surgical Sciences, Alma Mater Studiorum, University of Bologna 9296, Bologna, Italy; 3 Clinical Pharmacology Unit, IRCCS Azienda Ospedaliero-Universitaria di Bologna, Bologna, Italy; 4 Infectious Diseases Unit, IRCCS Azienda Ospedaliero-Universitaria di Bologna, Bologna, Italy; 5 Anesthesiology and Intensive Care Medicine, IRCCS Azienda Ospedaliero-Universitaria di Bologna, Bologna, Italy; 6 Division of Anesthesiology, Department of Anesthesia and Intensive Care, IRCCS Azienda Ospedaliero-Universitaria di Bologna, Bologna, Italy; Providence Portland Medical Center, Portland, Oregon, USA

**Keywords:** population pharmacokinetics, ceftolozane/tazobactam, PK/PD target attainment, ESBL-producing Enterobacterales

## Abstract

Ceftolozane/tazobactam is widely used against extended-spectrum β-lactamase (ESBL)-producing Enterobacterales, yet FDA-approved dosing relies on intermittent infusion and a fixed 2:1 ratio that assumes adequate β-lactamase inhibition. Tazobactam’s faster and variable clearance raises concerns for subtherapeutic exposure. This study evaluated whether continuous infusion (CI) improves pharmacokinetic target attainment and whether current dosing adequately covers both components. We conducted an analysis of 139 hospitalized adults receiving CI ceftolozane/tazobactam with therapeutic drug monitoring (TDM). Free steady-state concentrations (*f*Css) and observed clearance were calculated and assessed across kidney function strata. Population pharmacokinetic modeling and Monte Carlo simulations evaluated the probability of target attainment (PTA) for each drug. Despite kidney function-adjusted CI, 38.1% of patients had tazobactam *f*Css <4 mg/L, a threshold linked to reduced efficacy and resistance emergence, while only 2.5% had ceftolozane *f*Css <8 mg/L (namely 4× MIC the Enterobacterales clinical breakpoint for susceptibility). Tazobactam clearance (mean 7.41 L/h) was 2.5-fold higher than ceftolozane and highly variable (CV 115%). Kidney function explained only 23% of clearance variability. The cohort’s older age (median 66 years; 26.6% ≥75 years) likely led to lower tazobactam clearance, suggesting even greater underexposure in younger patients. Simulations showed standard dosing achieved only 29%–54% cumulative response against ESBL-producing Enterobacterales, versus 73%–82% with optimized regimens using 2–3× higher ceftolozane/tazobactam doses of magnitude similar to those licensed for pneumonia. Current ceftolozane/tazobactam dosing, even with CI, is often subtherapeutic for tazobactam. These findings challenge fixed-ratio formulations and support individualized, component-guided dosing to preserve efficacy and suppress resistance.

## INTRODUCTION

The emergence of extended-spectrum β-lactamase (ESBL)-producing Enterobacterales has radically reshaped our therapeutic approach to serious Gram-negative infections (1, 2). In this evolving landscape, ceftolozane/tazobactam stands out as a vital option, marrying a potent β-lactam with the β-lactamase inhibitor, tazobactam (3). In this paradigm, tazobactam underpins the uninterrupted efficacy of ceftolozane, shielding it from ESBL-mediated inactivation. Yet, prevailing PK/PD evaluations and therapeutic drug monitoring strategies may emphasize ceftolozane exposure, without accounting for tazobactam’s distinct pharmacokinetic behavior. Clinicians may not recognize that tazobactam clearance exceeds that of ceftolozane by approximately 3.5-fold (4–8). This imbalance in tazobactam exposure can threaten ceftolozane’s therapeutic effectiveness against ESBL-producing Enterobacterales, particularly in critically ill patients, where the fixed 2:1 dosing ratio based on its formulation may not consistently achieve the desired exposure.

For most β-lactams, the value of extended and continuous infusion (CI) in enhancing treatment efficacy is well established (9–11). Meta-analyses and guidelines consistently show that prolonged infusion of cefepime, piperacillin–tazobactam, and meropenem improves PK/PD target attainment and can reduce mortality, especially in sepsis or infections caused by resistant Gram-negative organisms (10, 12–14). Yet, despite ceftolozane/tazobactam’s similarly time-dependent killing, rigorous studies evaluating infusion strategies for this agent are scarce. A limited hollow-fiber model compared intermittent, extended, and continuous ceftolozane/tazobactam infusion regimens, and a small case series documented its safe use via CI in burn patients, but robust pharmacokinetic, clinical, and comparative data remain lacking (12, 15–17).

Crucially, consensus recommendations now emphasize that CI β-lactams should maintain drug concentrations above the MIC, and even several-fold above this at steady state for optimal outcomes (11). Yet, we lack clarity around tazobactam exposure: at what concentration does it effectively synergize with ceftolozane and may efficaciously prevent resistance development to ESBL-producing Enterobacterales, and can CI reliably maintain that target exposure, especially when tazobactam clearance is so high (18)?

This evidentiary gap underpins the rationale for our study. In a cohort of patients receiving CI ceftolozane/tazobactam, we systematically measured both drug components, assessed their *in vivo* exposure variability, and explored whether tazobactam concentrations can be predicted by clinical variables or ceftolozane concentrations. By bringing tazobactam into the PK/PD equation, we seek to ensure the “horse” (inhibitor) continues to pace the “cart” (the antibiotic) in order to preserve ceftolozane/tazobactam efficacy against ESBL-producing Enterobacterales.

## RESULTS

**Fig 1:**
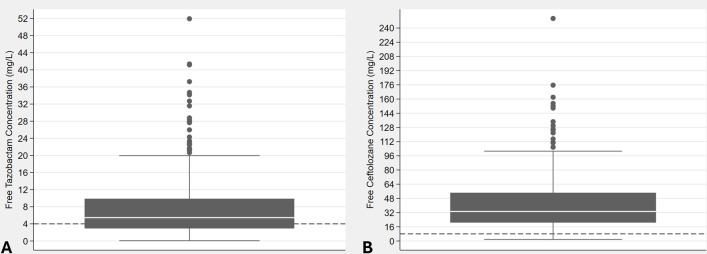
Box and whisker plot illustrating the distribution of (**A**) free tazobactam concentrations (38% of the observed concentrations below 4 mg/L) and (**B**) free ceftolozane concentrations (2.5% of the observed concentrations below 8 mg/L).

A total of 139 patients contributed 278 samples that were assayed to determine both steady-state concentrations of ceftolozane (Css_TOL_) and of tazobactam (Css_TAZ_). Demographic and clinical data are summarized in Table 1. Most patients were male (78/139, 56.1%). The population was generally older, with a median age of 66 years, and 26.6% of patients were 75 years of age or older. The median body weight was 70 kg, with a body mass index (BMI) of 25.7% with 18.7% of the population meeting the definition of obesity based on a BMI ≥30 kg/m^2^. Kidney function was estimated as both estimated glomerular filtration rate (eGFR) and estimated creatinine clearance (eCLcr), with a median value of 76 mL/min, when considering the product label, which is based on CLcr_WT. A median daily dose of 3.0 g/1.5 g of ceftolozane/tazobactam was used with a median free concentration of ceftolozane (*f*Css_TOL_) and of free concentration of tazobactam (*f*Css_TAZ_) of 33.3 and 5.46 mg/L, respectively. As shown in Fig. 1, a wide range (97.3%) coefficient of variation (CV) of *f*Css_TAZ_ was observed, and 38.1% of observed *f*Css_TAZ_ were below the free tazobactam concentration threshold (C_T_) of 4 mg/L. In contrast, the %CV of *f*Css_TOL_ was 78.8% and only 2.5% samples were below 8 mg/L (namely, four times the susceptibility breakpoint).

**TABLE 1:** Patient demographics, treatment, and exposure characteristics^*a*^

Characteristic	Value (*n* = 139 patients)
Demographic and clinical data	
Age (years)	66 (55–75)
Gender (male/female)	78/61
Body weight (kg)	70 (62–85)
BMI (kg/m^2^)	25.7 (22.5–29.0)
eGFR (mL/min/1.73 m^2^)	84.2 (39.4–105.8)
eGFR (mL/min)	85.7 (42.4–107.5)
eCLcr_WT (mL/min)	76.1 (42.4–117.6)
Ceftolozane/tazobactam treatment	
Median dose (g/daily)	3/1.5 (1.5/0.75–6/3)
*f*Css_TOL_ (mg/L)	33.3 (20.8–54.0)
*f*Css_TAZ_ (mg/L)	5.46 (2.96–9.85)
*f*Css_TOL_/*f*Css_TAZ_ ratio	6.3 (4.3–8.4)

^*a*^ Data are presented as median (IQR) for continuous variables and as number (%) for dichotomous variables. BMI, body mass index; eGFR, estimated glomerular filtration rate based on the 2021 Chronic Kidney Disease Epidemiology equation; eCLcr_WT, estimated creatinine clearance based on the Cockcroft-Gault equation; *f*Css_TOL_, free ceftolozane concentration; *f*Css_TAZ_, free tazobactam concentration.

**Fig 2:**
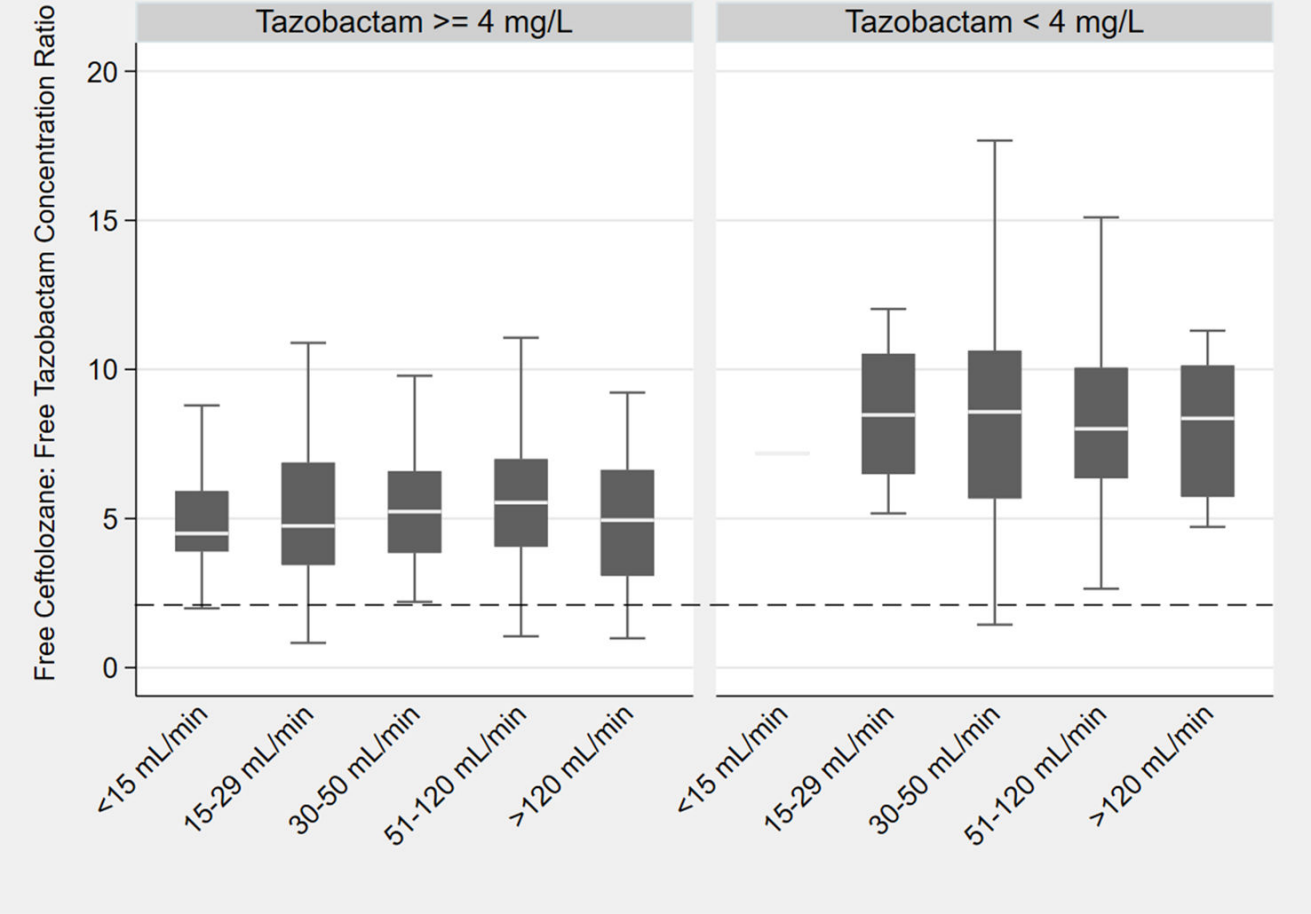
Box and whisker plot illustrating the distribution of free ceftolozane to free tazobactam concentration ratios, with higher ratios observed in patients with free tazobactam concentrations below 4 mg/L.

**Fig 3:**
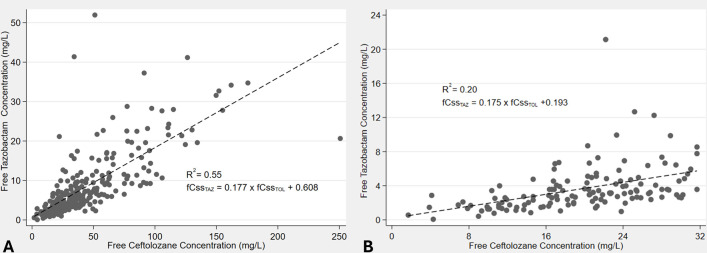
Scatter and linear fit plot of the free tazobactam concentrations to free ceftolozane concentrations across the range of observations (**A**) and restricted to free ceftolozane concentrations below 32 mg/L (**B**).

The mean (%CV) CL_TOL_obs_ and CL_TAZ_obs_ were 4.02 (119%) L/h and 10.6 (106%) L/h, respectively. The median CL_TOL_obs_ and CL_TAZ_obs_ were 3.08 and 7.27 L/h, respectively. Table 2 summarizes the univariate relationships of key clinical variables and CL_TAZ_obs_ in order to help predict scenarios for low (<4 mg/L) *f*Css_TAZ_. As shown, age, weight, height, and body surface area (BSA) were poor predictors of tazobactam clearance (CL_TAZ_). In relative terms, kidney function was a better predictor with eGFR in mL/min, performing the best based on the coefficient of determination. However, as is known for many antibiotics, kidney function only accounted for 23% of the interindividual variability in CL_TAZ_. The strongest correlation was observed between observed ceftolozane clearance (CL_TOL_obs_) and observed tazobactam clearance (CL_TAZ_obs_) (R^2^ = 0.42). The median *f*Css_TOL_:*f*Css_TAZ_ was 6.5 with no clear relationship across eCLcr, though higher values were noted when *f*Css_TAZ_ was <4 mg/L (Fig. 2). To better predict this risk for low *f*Css_TAZ_, regression analyses (Fig. 3A) identified *f*Css_TOL_ as the best predictor of this relationship (R^2^ = 0.55). However, this translation is less predictive (R^2^ = 0.20) at the lower end of this distribution, i.e., the likelihood of *f*Css_TAZ_ <4 mg/L is poorly predicted by a low (<32 mg/L) measurement of *f*Css_TOL_ (Fig. 3B).

**TABLE 2:** Univariate relationship of the observed tazobactam clearance (CL_TAZ_obs_) to key demographic variables, kidney function estimation methods, and ceftolozane clearance (CL_TOL_obs_)^*a*^

Variable	R^2^	Slope^*b*^	Constant^*b*^
Age (year)	0.0040	−0.009 [−0.08, 0.06]	11.2 [6.72, 15.7]
Weight (kg)	0.025	0.075 [0.02, 0.13]	5.08 [0.82, 9.34]
Height (m)	0.001	0.041 [−0.10, 0.18]	3.71 [−19.8, 27.3]
BSA (m^2^)	0.022	5.15 [1.03, 9.26]	1.27 [−6.29, 8.84]
SCr (mg/dL)	0.10	−2.03 [−2.74, −1.32]	13.6 [12.0, 15.1]
CLcr_WT (mL/min)	0.16	0.060 [0.044, 0.076]	5.51 [3.75, 7.27]
eGFR (mL/min/1.73 m^2^)	0.13	0.090 [0.063, 0.15]	3.82 [1.46, 6.18]
eGFR (mL/min)	0.23	0.12 [0.096, 0.15]	1.26 [−1.03, 3.54]
CL_TOL_obs_ (L/h)	0.42	2.02 [1.74, 3.32]	2.98 [1.60, 4.36]

^*a*^ BSA, body surface area based on the DuBois-DuBois method; SCr, serum creatinine; CLcr, creatinine clearance based on the Cockcroft-Gault equation; eGFR based on the 2021 Chronic Kidney Disease Epidemiology equation.

^*b*^ Values reported as coefficient [95% confidence interval].

Similar to the findings of the observed CL estimates, the population pharmacokinetic estimates mean (%CV) of population ceftolozane clearance (CL_TOL_pop_) and of population tazobactam clearance (CL_TAZ_pop_) were 2.96 (93%) L/h and 7.41 (115%) L/h, respectively (Table S2). Statistical testing within Monolix identified strong correlations between CL_TOL_pop_ and CL_TAZ_pop_, and kidney function estimates, particularly with eGFR compared to eCLcr (Tables S3 and S4). Stepwise comparisons between each kidney function estimation model and CL_TOL_ and CL_TAZ_ are provided in Table S5. As shown, kidney function based on the 2021 CKD-EPI equation, EKFC, eCLcr_LBW, and eCLcr_DW, all based on mL/min, were indistinguishable based on Akaike information criteria (AIC). The final model was selected based on the 2021 CKD-EPI equation in mL/min, given calls to harmonize this approach by the National Kidney Foundation (19). The final model parameters are included in Table S6 with diagnostic plots included (Fig. S2 to S6). Simulations for the probability of target attainment (PTA) estimation were performed based on kidney function (eGFR) groups with distributions to match patient characteristics (Table S7). Table 3 provides the PTA of aggressive joint PK/PD target and the cumulative fraction of response (CFR) against ESBL-producing Enterobacterales for ceftolozane/tazobactam. As shown, the current approved label dosing achieves only quasi-optimal PTA of ceftolozane/tazobactam, and the CFR was always significantly suboptimal against ESBL-producing Enterobacterales (ranging 29%–54%). Approximately 2.3-fold higher daily doses having a magnitude similar to those licensed for pneumonia are projected to be necessary to achieve in most classes of renal function CFR against ESBL-producing Enterobacterales isolates that are at least quasi-optimal (ranging 73%–82%).

**TABLE 3:** PTA of aggressive joint PK/PD target and CFR for ceftolozane (TOL)/tazobactam (TAZ) against ESBL-producing Enterobacterales dosage regimens across different eGFR classes^*a*^

	eGFR (mL/min)	Daily TOL/TAZ dose (g)	TAZ	TOL								CFR
			C_T_	MIC								
			4	0.25	0.50	1	2	4	8	16	32	
Current product label	<15	0.30/0.15	50.1	100	99.9	98.1	87.4	61.0	17.7	0.2	0	43
	15–29	0.70/0.375	40.3	100	100	99.5	90.3	56.3	16.3	0.7	0	35
	30–50	1.5/0.75	54.6	100	100	99.8	96.0	72.1	30.1	3.1	0	48
	51–120	3.0/1.5	60.1	100	100	99.9	97.5	80.9	39.4	5.6	0.5	54
	>120	3.0/1.5	33.5	100	100	99.4	93.7	62.1	18.9	1.9	0	29
Suggested alternate regimen	<15	0.70/0.375	80.5	100	100	100	99.0	90.0	56.1	8.8	0	73
	15–29	2.0/1.5	87.7	100	100	100	99.6	97.0	71.1	24.5	0.9	80
	30–50	3.0/1.5	86.5	100	100	100	99.8	96.0	71.9	28.7	2.2	80
	51–120	6.0/3.0	88.6	100	100	100	99.9	97.5	80.9	39.2	5.4	82
	>120	6.0/3.0	82.5	100	100	100	99.7	96.5	71.7	27.7	4.1	76

^*a*^ Values reported as percent probability of target attainment based on *f*Css_TAZ_/C_T_ ratio ≥1 and *f*Css_TOL_/MIC ratio ≥4, where *f*Css_TAZ_ is the free tazobactam concentration; C_T_ is the tazobactam concentration threshold; *f*Css_TOL_ is the free ceftolozane concentration; and MIC is the minimum inhibitory concentration. $CFR=\sum_{i=1}^{n}{PTA_{-}CT}_{TAZ}\times {PTA_{-}MIC}_{TOL}\times fiMIC_{i}$, where *n* is the number of discrete MIC values, PTA_CT_TAZ_ is the probability of target attainment for tazobactam, PTA_MIC_TOL_ is the probability of target attainment for ceftolozane at the discrete MIC, and *fi* is the fraction of isolates at the MIC. The daily dose was after a 3 g loading dose (1 h infusion) with PTA determined at 72 h (3 days of CI of the daily dose). Shaded regions highlight PTA ≥ 90% for TOL and CFR ≥ 80%.

## DISCUSSION

This study highlights a critical and underappreciated vulnerability in the clinical use of ceftolozane/tazobactam despite CI, an administration strategy designed to optimize the PK/PD for time-dependent agents. Tazobactam concentrations frequently fell below the pharmacodynamic threshold associated with effective ESBL inhibition for preventing resistance development to ceftolozane–tazobactam (17, 20, 21). While ceftolozane concentrations reliably exceeded standard PK/PD targets (e.g., *f*T > 4× MIC), 38% of measured tazobactam steady-state levels were <4 mg/L, a threshold supported by *in vitro* and PK/PD studies as necessary for suppressing resistance development by ESBL-producing Enterobacterales. In this latter regard, it was shown that the target required for the prevention of emergence of resistance to ceftolazone–tazobactam by ESBL-producing Enterobacterales is much greater than that required for the static effect or the 1−log10 reduction associated with efficacy (21). Conversely, targeting *f*T >MIC of ceftolozane at 100% 4× MIC in the presence of 4 mg/L of tazobactam prevented the risk of emergence of resistance (21). This gap between ceftolozane and tazobactam exposure challenges the long-standing assumption embedded in current FDA labeling that fixed 2:1 ratio dosing based on intermittent infusion ensures adequate inhibitory activity. In contrast, our study employed CI, the most pharmacologically rational approach for β-lactams, yet still revealed significant underexposure of tazobactam (9–11). These findings underscore that even under optimized delivery conditions, the current dosing framework is likely insufficient, particularly in high-risk patients.

Our pharmacokinetic data confirm that tazobactam clearance (CL_TAZ_) exceeds that of ceftolozane (CL_TOL_) by a factor of three, a finding consistent with prior studies (4–8). This was demonstrated with both population PK modeling and without model-based assumptions. Importantly, our observed CL_TAZ_ values were lower than previously reported across the globe (Table 4), a discrepancy explained by the advanced age of our cohort, nearly one-quarter of whom were over 75 years old. Reduced kidney function in older patients likely attenuated drug clearance, partially mitigating tazobactam underexposure. This raises a critical concern in younger patients with preserved or augmented kidney function, in whom tazobactam underdosing may be even more pronounced, with important implications for treatment failure and resistance emergence. Multiple newly approved β-lactam antibiotics (ceftobiprole, sulbactam-durlobactam, cefedirocol, etc.) include recommendations for dose adjustment in the situation of kidney function >120–130 mL/min that is presently not included with ceftolozane/tazobactam’s label.

**TABLE 4:** Summary of the mean population characteristics, estimates of clearance, and ratio of tazobactam to ceftolozane clearance in the current study compared to others published in the literature^*a*^

Source	*N*	Age (years)	Weight (kg)	CLcr (mL/min)	CL_TOL_ (L/h)	CL_TAZ_ (L/h)	CL ratio (CL_TAZ_:CL_TOL_)
Current study	139	66	70.0	76	2.96	7.41	2.52
Chandorkar et al. (4)	367	45	73.5	109	5.11	18	3.52
Monogue et al. (5)	20	25	53.2	118	4.57	20.5	4.49
Sime et al. (6)	12	56	80	108	7.20	25.4	3.53
Liu et al. (7)	12	27	61.8	NR	6.09	20.8	3.42
Kakara et al. (8)	184	68	59.4	74	5.04	15.5	3.08

^*a*^ CLcr, creatinine clearance; CL_TOL_, ceftolozane clearance; CL_TAZ_, tazobactam clearance.

Further complicating dosing precision, kidney function accounted for only 23% of the variability in CL_TAZ_. Other unexplained sources, such as tubular secretion, non-renal elimination, protein binding alterations, and fluid shifts, likely drive the wide interindividual variability observed. While CL_TOL_ correlated modestly with CL_TAZ_ (R^2^ = 0.42), this relationship broke down at lower drug exposures precisely where clinicians are most vulnerable to unseen underdosing.

The pharmacodynamic consequences of tazobactam underexposure against ESBL-producing Enterobacterales in terms of risk of resistance emergence are well supported by experimental data. Soon et al. showed that ceftolozane activity was enhanced by the addition of tazobactam in a concentration-dependent manner against all beta-lactamase-producing strains and that when tazobactam concentrations fell below 4 mg/L, ceftolozane EC_50_ values rose significantly, particularly at higher bacterial inocula, leading to delayed or absent bactericidal activity (20). Similarly, Wi et al. demonstrated that 96% of ESBL-producing *Escherichia coli* and *Klebsiella pneumoniae* isolates may exhibit an inoculum effect when tested in the presence of a fixed tazobactam concentration of 4 mg/L. Interestingly, a 4- to 64-fold MIC increase was observed in the presence of high bacterial loads (10⁷ CFU/mL) compared to low bacterial loads (10^5^ CFU/mL) (22). These data align with our findings and raise concern for underperformance in real-world infections, especially when characterized by high organism burden, such as ventilator-associated pneumonia or bloodstream infections.

Susceptibility data further illuminate the risks of relying on static dosing. In a national French study, Jousset et al. showed that while CTX-M-producing Enterobacterales were generally susceptible to ceftolozane/tazbactam, only 13% of SHV-producing isolates retained susceptibility (23). Similarly, Castanheira et al. found diminished ceftolozane/tazobactam susceptibility among *K. pneumoniae* and *Enterobacter cloacae* relative to *E. coli*, despite carrying similar ESBL genotypes (24). These data emphasize that tazobactam’s efficacy varies by organism and resistance mechanism, a nuance poorly accounted for by fixed-ratio dosing.

Taken together, our findings suggest a paradigm shift in how clinicians approach the dosing of β-lactam/β-lactamase inhibitor combinations. Relying on ceftolozane exposure alone may offer a false sense of security. In the context of resistance suppression and treatment success, tazobactam must be explicitly targeted in ESBL-producing Enterobacterales infections. Precision dosing strategies, including decoupled ratio administration, higher fixed tazobactam doses, or model-informed precision dosing using real-time therapeutic drug monitoring (TDM), may be essential to avoid unintended underdosing in critical infections.

Our study has limitations. It was retrospective and single-center. Protein binding assumptions may not fully account for pathophysiologic variation in critically ill patients. We also relied on eGFR rather than measurement of kidney function, such as 24-h creatinine clearance, that may not account for variation in critically ill patients. Microbiologic and clinical outcomes were not uniformly available, limiting linkage between exposure and treatment success. Nonetheless, strengths include dual-analyte quantification with validated liquid chromatography-tandem mass spectrometry (LC-MS/MS), component-specific clearance estimates under steady-state CI conditions, and inclusion of a real-world, older patient cohort that is often excluded from registration trials but is highly relevant to clinical practice.

Despite optimized delivery via CI, current ceftolozane/tazobactam dosing frequently fails to achieve adequate tazobactam exposure against ESBL-producing Enterobacterales. These findings call into question the adequacy of fixed-ratio regimens, particularly in younger, renally competent patients or in infections with high bacterial burden or for preventing resistance development to ceftolozane/tazobactam by ESBL-producing Enterobacterales. Future research should define validated pharmacodynamic targets for tazobactam and assess whether individualized, exposure-guided dosing improves outcomes. Our results challenge the conventional wisdom that a potent β-lactam is sufficient if the β-lactamase inhibitor is simply “along for the ride.” In this partnership, tazobactam is the horse clearing the way by inhibiting ESBL, while ceftolozane is the cart, delivering the bactericidal payload. If the horse falters, the cart cannot move forward. To treat ESBL-producing Enterobacterales effectively, we must keep the horse with the cart.

## MATERIALS AND METHODS

### Study design and patient population

This retrospective cohort study included adult patients admitted to the IRCCS Azienda Ospedaliero-Universitaria di Bologna (Italy) between November 2023 and June 2025 who received CI ceftolozane–tazobactam for treatment of Gram-negative infections and underwent real-time TDM of both agents.

Initial ceftolozane/tazobactam dosing consisted of a 2 g/1 g loading dose infused over 1 h, followed immediately by CI maintenance dosing adjusted for eGFR:

- ≥50 mL/min/1.73 m²: 6 g/3 g daily
- 30–49 mL/min/1.73 m²: 3 g/1.5 g daily
- 15–29 mL/min/1.73 m²: 1.5 g/0.75 g daily
- <15 mL/min/1.73 m²: 0.9 g/0.45 g daily

Doses were subsequently refined through TDM-guided clinical pharmacology consultation. Steady-state plasma concentrations for ceftolozane (Css_TOL_) and tazobactam (Css_TAZ_) were measured 48–72 h after therapy initiation and re-assessed when feasible. Quantification was performed using a validated LC-MS/MS assay (lower limits of quantification: 0.2 mg/L for ceftolozane, 0.1 mg/L for tazobactam) (25). Free concentrations (*f*Css_TOL_*, f*Css_TAZ_) were estimated using free-fractions correction factors of 0.81 and 0.70, respectively (26).

Clinical and laboratory data extracted from the medical record included age, sex, weight, height, serum creatinine, ceftolozane/tazobactam dosing, and comorbidities. Kidney function was estimated using the 2021 CKD-EPI equation (with and without BSA indexation) and the EKFC equation (with and without BSA indexation) for eGFR (27, 28). eCLcr was generated using the Cockcroft-Gault equation (29). Since the current label for ceftolozane is based on eCLcr and weight (eCLcr_WT), we also tested the use of lean body weight (LBW), ideal body weight (IBW), adjusted body weight, and dosing weight (DW) to account for obesity (30). The dosing weight is based on the use of weight when less than IBW, adjusted body weight when weight ≥1.25 × IBW, or use of IBW when these conditions are not met. A complete list (codebook) of evaluated variables and estimation equations for kidney function is provided in Table S1. Patients receiving renal replacement therapy were excluded. Observed ceftolozane clearance (CL_TOL_obs_) and tazobactam clearance (CL_TAZ_obs_) were estimated by use of the standard equation CL = Rate of Infusion (mg/h)/Steady state concentration (C_ss_), for each compound (Css_TAZ_, Css_TOL_), respectively.

### Population pharmacokinetic modeling

Ceftolozane/tazobactam plasma concentrations were analyzed by means of non-linear mixed-effect modeling using the stochastic approximation expectation minimization algorithm provided by the Monolix software (version 2024R1; Lixoft, Antony, France). Since all subjects received CI ceftolozane/tazobactam administration, a one-compartment model for fitting simultaneously both ceftolozane and tazobactam concentrations was created. The first step was a linear model (base model) parameterized with zero-order administration and CL from each drug-specific compartment, one for ceftolozane (CL_TOL_) and the other for tazobactam (CL_TAZ_), as shown in Fig. S1. Volumes of distribution (V) of both ceftolozane and tazobactam were fixed based on prior population PK models, namely 32.1 and 43.3 L, respectively, as explained in Fig. S1 (31). All individual parameters were considered to be log-normally distributed. Several error models (additive, proportional, or a combined additive and proportional error model) were tested for residual variability.

In the second step, the impact of different covariates, such as age, body weight, and kidney function, on the base model was assessed. Covariates were selected according to a forward/backward process. In the forward step, the inclusion of a covariate in the model was based on the result of Pearson’s correlation test between each covariate and the random effect of the estimated pharmacokinetic parameter.

### Model selection and validation

The diagnostic criteria for comparing the different models and for identifying which significant covariates had to be included in the final model were a reduction of the objective function value >3.82 points and of both the AIC and the Bayesian Information Criteria by at least two points. The adequacy of the different models was also assessed by considering the goodness of fit of the observed versus predicted concentrations and the relative standard error of the pharmacokinetic parameter estimates. Visual predictive check showing the time course of the 10th, the 50th, and the 90th percentiles of observed data overlaid with the corresponding 90% prediction intervals was used for internal validation.

### Monte Carlo simulation

One thousand-subject Monte Carlo simulations were performed using the final population pharmacokinetic model by means of Simulx 2024R1 (Lixoft, Antony, France) in order to generate different pharmacokinetic concentration-time profiles associated with different dosing regimens of CI ceftolozane/tazobactam in relation to five classes of renal function. The simulated daily regimens of ceftolozane/tazobactam delivered by CI were tested based on the approved label dosing as the total daily dose with the inclusion of a 2 g/1 g loading dose (1 h infusion) prior to CI. We also tested alternate ceftolozane/tazobactam daily doses (in units of 1 g/0.5 g based on vial size) to identify regimens that could achieve high enough PTA of aggressive joint PK/PD targets of *f*Css_TOL_/MIC ratio ≥4 and *f*Css_TAZ_/C_T_ ≥1 against ESBL-producing Enterobacterales (32). The tested MIC distribution ranged between 0.25 and 32 mg/L for ceftolozane, and the target concentration (C_T_) for tazobactam was 4 mg/L, namely the fixed target concentration threshold used by the EUCAST for testing the *in vitro* standard susceptibility of TOL/TAZ against Enterobacterales. This aggressive target was defined because it was deemed effective in preventing resistance development to ceftolozane–tazobactam by ESBL-producing Enterobacterales, similar to what our group had previously established for other β-lactam/β-lactamase inhibitor combinations (33, 34). In this regard, *in vitro* experimental models showed that the target required for the prevention of emergence of resistance to ceftolazone–tazobactam by ESBL-producing Enterobacterales was much greater than that required for a static effect or a 1−log10 reduction and may also increase as the time of exposure increases (21). Interestingly, in the presence of 4 mg/L of tazobactam, the maximum risk of resistance generally occurred at a *f*T >MIC of ceftolozane around 30%, which is similar to the static target of *f*T >MIC (21). Conversely, emergence of resistance did not occur at an *f*T >MIC of 100% 4× MIC of ceftolozane (21).

The PTAs were considered optimal when both the likelihood of attaining *f*Css_TOL_/C_T_ ≥1 and *f*Css_TOL_/MIC ratio ≥4 were ≥90%, quasi-optimal if only one of the two thresholds was ≥90% and suboptimal if neither of the two thresholds was ≥90%. The CFR achievable with the selected ceftolozane/tazobactam dosing regimens delivered by CI was calculated against the MIC distribution (0.25 to 32 mg/L) of an ESBL collection of Enterobacterales isolates (*n* = 169) (23). The CFRs were considered optimal if >90%, quasi-optimal if between 80% and 90%, and suboptimal if <80% (35).

### Statistical analyses

Descriptive statistics were used to characterize patient demographics, treatment, and exposure profiles. Box and whisker plots were used to visualize the central tendency profiles of drug concentrations and their ratios across kidney function. Ordinary least squares regression was used to characterize the relationship between CL_TAZ_ and *f*Css_TAZ_ and key variables.

## SUPPLEMENTAL MATERIAL

Supplemental material aac.01215-25-s0001.docx Tables S1 to S7; Fig. S1 to S6.

ASM does not own the copyrights to Supplemental Material that may be linked to, or accessed through, an article. The authors have granted ASM a non-exclusive, world-wide license to publish the Supplemental Material files. Please contact the corresponding author directly for reuse.
