# Supplementary material for: Keeping the horse with the cart: are we underdosing tazobactam even when using continuous-infusion ceftolozane/tazobactam for effectively preventing resistance development by ESBL-producing Enterobacterales?
Source: Antimicrob Agents Chemother. 2025 Oct 29;69(12):e01215-25. doi: 10.1128/aac.01215-25 (PMC12691610; doi:10.1128/aac.01215-25)

**Keeping the Horse with the Cart: Are We Underdosing Tazobactam Even When Using Continuous-Infusion Ceftolozane/Tazobactam Against ESBL-producing Enterobacterales?**

Manjunath P. Pai , Pier Giorgio Cojutti, Milo Gatti, Matteo Rinaldi, Tommaso Tonetti, Antonio Siniscalchi, Pierluigi Viale, Federico Pea

**Supplemental File Contents**

**TABLES**

**Table S1.** Body size descriptors and kidney function estimation equations used in this study

**Table S2.** Parameter estimates of the base pharmacokinetic model

**Table S3.** Univariate analyses to identify significant covariates on CL_TOL_

**Table S4.** Univariate analyses to identify significant covariates on CL_TAZ_

**Table S5.** Comparison of different kidney function equations as covariates of CL with the best identified structural model based on the greatest reduction in the Akaike Information Criterion (AIC) value and confidence in parameter estimates

**Table S6.** Parameter estimates of the final pharmacokinetic model

**Table S7.** Distribution of participants across estimated glomerular filtration rate groups including central tendency and variance of the population to support simulations

**FIGURES**

**Figure S1.** Structure of the base pharmacokinetic model for ceftolozane

**Figure S2**. Ceftolozane (A) and Tazobactam (B) population-predictions vs. observations and individual-predictions vs. observations for the final population pharmacokinetic model

**Figure S3**. Scatter plot of the residual vs. time and of the residual vs. individual predictions of ceftolozane (A) and tazobactam (B) for the final population pharmacokinetic model

**Figure S4**. Distribution of the Individual Weighted Residuals and of the Normalized Prediction Distribution Errors of ceftolozane (A) and tazobactam (B) for the final population pharmacokinetic model

**Figure S5**. Visual predictive check (VPC) for the ceftolozane (A) and tazobactam(B) final population pharmacokinetic model

**Figure S6**. Distribution of the individual parameters

**Table S1.** Body size descriptors and kidney function estimation equations used in this study

| Descriptor | Full Name | Unit |
| --- | --- | --- |
| wt | Weight | Weight in kg |
| ht | Height | Height in cm |
| bmi | Body mass index | Computed in kg/m^2^ |
| ibw | Ideal body weight | Estimated in kg |
| adjbw | Adjusted body weight | Estimated in kg |
| dw | Dosing weight | Estimated in kg |
| lbw | Lean body weight | Estimated in kg |
| bsa_m | Body surface area using Mosteller’s adaptation | Estimated in m^2^ |
| bsa_dd | Body surface area using Dubois-DuBois equation | Estimated in m^2^ |
| clcr_wt | Creatinine clearance using the Cockcroft-Gault equation and weight | Estimated in mL/min |
| clcr_ibw | Creatinine clearance using the Cockcroft-Gault equation and ideal body weight | Estimated in mL/min |
| clcr_adjbw | Creatinine clearance using the Cockcroft-Gault equation and adjusted body weight | Estimated in mL/min |
| clcr_dw | Creatinine clearance using the Cockcroft-Gault equation and dosing weight | Estimated in mL/min |
| ckdepi2021 | Glomerular filtration rate using the Chronic Kidney Disease Epidemiology Equation without race published in 2021 | Estimated in mL/min/1.73 m^2^ |
| ckdepi2021mlmin_bsa_m | Glomerular filtration rate using the Chronic Kidney Disease Epidemiology Equation without race published in 2021 normalized to individual body surface area- Mosteller equation | Estimated in mL/min |
| ckdepi2021mlmin_bsa_dd | Glomerular filtration rate using the Chronic Kidney Disease Epidemiology Equation without race published in 2021 normalized to individual body surface area-Dubois Dubois equation | Estimated in mL/min |
| scr_q | Serum creatinine normalized values for EKFC equation | unitless |
| ekfc | European Kidney Function Consortium (EKFC) equation | Estimated in mL/min/1.73 m^2^ |
| ekfc_mlmin_bsa_m | Glomerular filtration rate using the EKFC equation without race published in 2022 normalized to individual body surface area- Mosteller equation | Estimated in mL/min |
| ekfc_mlmin_bsa_dd | Glomerular filtration rate using the EKFC equation without race published in 2022 normalized to individual body surface area- Dubois-DuBois equation | Estimated in mL/min |

**Equations**

The formulas used to compute key variables are included below based on code used in STATA

****body weight**

gen ibw = 45.5 + 2.3*((ht/2.54)-60)

replace ibw = ibw + 4.5 if sex=="Male"

gen adjbw = 0.4*(wt-ibw) + ibw

gen dw= ibw

replace dw = wt if wt<ibw

replace dw = adjbw if wt>=1.25*ibw

gen bsa_m = ((wt*ht)/3600)^0.5

gen bsa_dd = (ht^0.725)*(0.007184)* (wt)^0.425

gen lbw = (9270*wt)/(6680+216*bmi)

replace lbw = (9270*wt)/(8780+244*bmi) if sex=="Female"

****CLcr**

gen CLcr_wt = (140-age)*wt/(72*scr)

replace CLcr_wt = CLcr_wt * 0.85 if sex=="Female"

gen CLcr_ibw = (140-age)*ibw/(72*scr)

replace CLcr_ibw = CLcr_ibw * 0.85 if sex=="Female"

gen CLcr_adjbw = (140-age)*adjbw/(72*scr)

replace CLcr_adjbw = CLcr_adjbw * 0.85 if sex=="Female"

gen CLcr_dw = (140-age)*dw/(72*scr)

replace CLcr_dw = CLcr_dw * 0.85 if sex=="Female"

gen CLcr_lbw = (140-age)*lbw/(72*scr)

****GFR**

*CKD EPI 2021

gen CKDEPI2021 = 142*((scr/0.7)^-0.242)*(0.9938^age)*1.012 if sex=="Female" & scr<=0.7

replace CKDEPI2021 = 142 * ((scr/0.7)^-1.200) * (0.9938^age) * 1.012 if sex=="Female" & scr>0.7

replace CKDEPI2021 = 142 * ((scr/0.9)^-0.302) * (0.9938^age) if sex=="Male" & scr<=0.9

replace CKDEPI2021 = 142 * ((scr/0.9)^-1.200)* (0.9938^age) if sex=="Male" & scr>0.9

gen CKDEPI2021mlmin_bsa_m = CKDEPI2021*bsa_m/1.73

gen CKDEPI2021mlmin_bsa_dd = CKDEPI2021*bsa_dd/1.73

*EKFC (European Kidney Function Consortium)-

gen scr_q = scr/0.9 if sex=="Male"

replace scr_q = scr/0.7 if sex=="Female"

gen ekfc = 107.3*scr_q^-0.332 if age<=40 & scr_q<1 & sex=="Female"

replace ekfc = 107.3*scr_q^-1.132 if age<=40 & scr_q>=1 & sex=="Female"

replace ekfc = 107.3*scr_q^-0.332 if age<=40 & scr_q<1 & sex=="Male"

replace ekfc = 107.3*scr_q^-1.132 if age<=40 & scr_q>=1 & sex=="Male"

replace ekfc = 107.3*(scr_q^-0.332)*0.990^(age-40) if age>40 & scr_q<1

replace ekfc = 107.3*(scr_q^-1.132)*0.990^(age-40) if age>40 & scr_q>=1

*EKFC (European Kidney Function Consortium)- in ml/min

gen ekfc_mlmin_bsa_m = ekfc*bsa_m/1.73

gen ekfc_mlmin_bsa_dd = ekfc*bsa_dd/1.73

**Figure S1.** Structure of the base pharmacokinetic model for ceftolozane


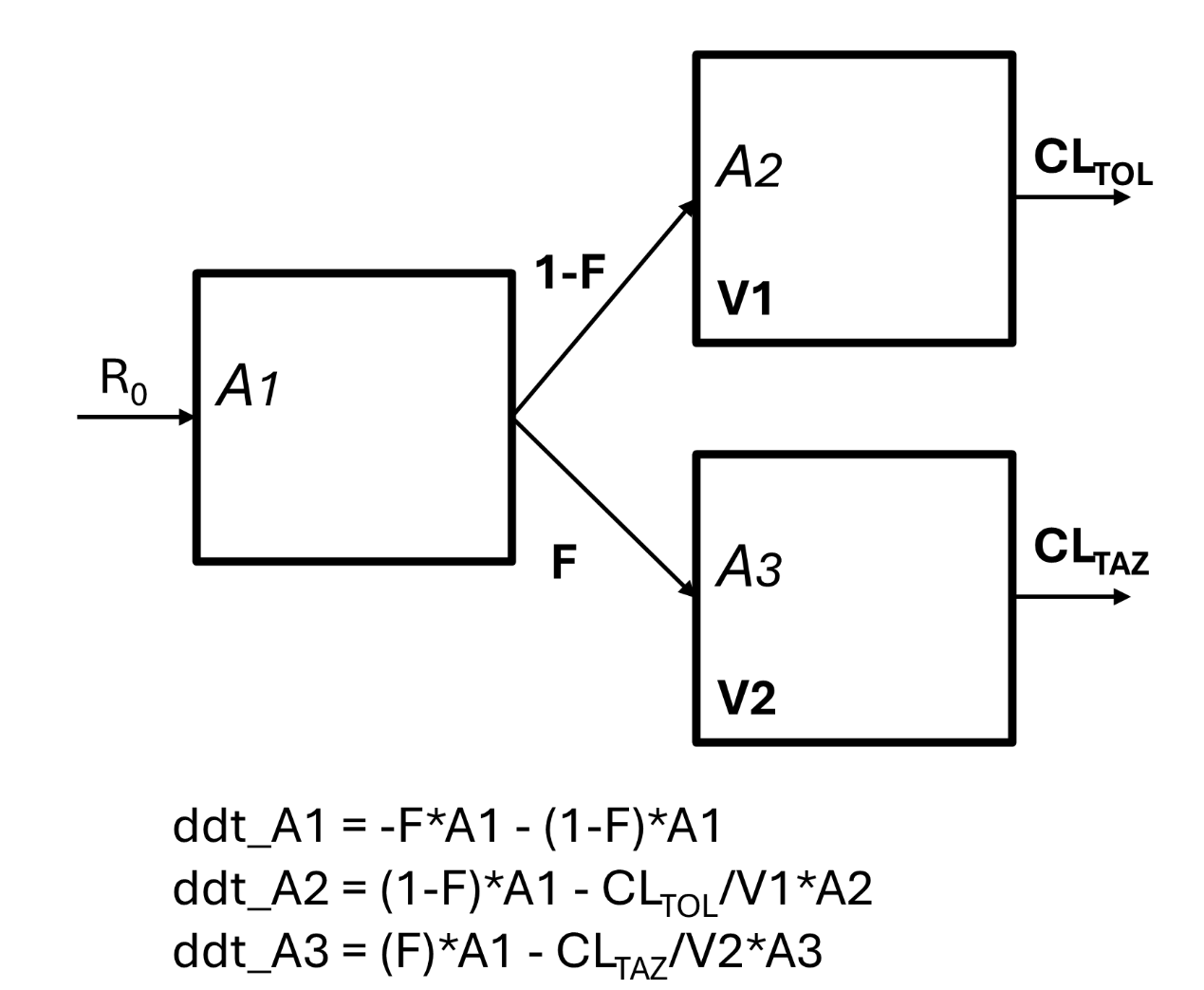


**Initial Parameter Estimates**

| Mean Parameter Estimates | Pop PK (Merck &Co, Inc)* |
| --- | --- |
| CL_TOL_ (L/h) | 5.4 |
| V_TOL_ (L) | 32.1 |
| CL_TOL_ (L/h) | 17.3 |
| V_Taz_ (L) | 43.3 |
| F | 0.333 |

*Caro L, Larson KB, Nicolau DP, De Waele J, Kuti JL, Saralaya R, Gadzicki E, Adedoyin A, Zeng Z and Rhee EG, P2225 ECCMID 21-24 April, 2018, Madrid, Spain, and study report of MK-7625A PN007 (data on file at Merck & Co., Inc., Kenilworth, New Jersey, USA). Ceftolozane/tazobactam 3000 mg (ceftolozane 2000 mg/tazobactam 1000 mg) or adjusted based on CrCL q8h by IV infusion over 1 hour in ventilated patients with suspected or confirmed pneumonia. [EUCAST rationale document Ceftolozane/tazobactam Version 1.0 Page 6 of 16]. F represented in this model was set based on the known formulation fraction of ceftolozane : tazobactam of 2:1.

**Table S2.** Parameter estimates of the base pharmacokinetic model

|  | **VALUE** | | **STOCH. APPROX.** | | | |
| --- | --- | --- | --- | --- | --- | --- |
|  |  |  | **S.E.** | **R.S.E.(%)** | **P2.5** | **P97.5** |
| **Fixed Effects** | | | | | | |
| **V1_pop** | 32.1 | |  |  |  |  |
| **Cltol_pop** | 2.96 | | 0.21 | 7.25 | 2.57 | 3.41 |
| **Cltaz_pop** | 7.41 | | 0.65 | 8.82 | 6.24 | 8.8 |
| **V2_pop** | 43.3 | |  |  |  |  |
| **F_pop** | 0.33 | |  |  |  |  |
| **Standard Deviation of the Random Effects** | | | | | | |
|  | **Value** | **C.V.(%)** |  |  |  |  |
| **omega_V1** | 0.42 | 43.92 |  |  |  |  |
| **omega_Cltol** | 0.79 | 93.41 | 0.055 | 6.97 | 0.69 | 0.91 |
| **omega_Cltaz** | 0.92 | 115.19 | 0.071 | 7.76 | 0.79 | 1.07 |
| **omega_V2** | 0.38 | 39.41 |  |  |  |  |
| **Error Model Parameters** | | | | | | |
| **a1** | 7.41 | | 0.97 | 13.0 | 5.76 | 9.53 |
| **b1** | 0.15 | | 0.027 | 17.6 | 0.11 | 0.21 |
| **a2** | 1.32 | | 0.25 | 19.1 | 0.92 | 1.91 |
| **b2** | 0.34 | | 0.033 | 9.77 | 0.28 | 0.41 |

**Table S3.** Univariate analyses to identify significant covariates on CL_TOL_

| **Cl_TOL_** | **COEFF** | **STATISTICS** | **P-VALUE** |
| --- | --- | --- | --- |
| sex |  | 1.03 | 3.13E-01 |
| adjbw | 0.17 | 2.01 | 4.60E-02 |
| age | 0.015 | 0.17 | 8.62E-01 |
| bmi | 0.13 | 1.49 | 1.37E-01 |
| bsa_dd | 0.19 | 2.3 | 2.29E-02 |
| bsa_m | 0.19 | 2.28 | 2.41E-02 |
| ckdepi2021 | 0.43 | 5.59 | 1.19E-07 |
| ckdepi2021mlmin_bsa_dd | 0.57 | 8.13 | 2.29E-13 |
| ckdepi2021mlmin_bsa_m | 0.57 | 8.16 | 1.90E-13 |
| clcr_adjbw | 0.41 | 5.34 | 3.83E-07 |
| clcr_dw | 0.49 | 6.64 | 6.79E-10 |
| clcr_ibw | 0.32 | 3.91 | 1.47E-04 |
| clcr_lbw | 0.5 | 6.74 | 3.98E-10 |
| clcr_wt | 0.47 | 6.28 | 4.26E-09 |
| dw | 0.17 | 2.02 | 4.56E-02 |
| ekfc | 0.45 | 5.87 | 3.14E-08 |
| ekfc_mlmin_bsa_dd | 0.56 | 8 | 4.65E-13 |
| ekfc_mlmin_bsa_m | 0.56 | 8.01 | 4.53E-13 |
| ht | 0.11 | 1.33 | 1.85E-01 |
| ibw | 0.11 | 1.35 | 1.80E-01 |
| lbw | 0.17 | 2 | 4.76E-02 |
| scr | -0.46 | -6.05 | 1.29E-08 |
| wt | 0.17 | 2 | 4.73E-02 |

**Table S4.** Univariate analyses to identify significant covariates on CL_TAZ_

| **Cl_TAZ_** | **COEFF** | **STATISTICS** | **P-VALUE** |
| --- | --- | --- | --- |
| sex |  | 0.44 | 5.09E-01 |
| adjbw | 0.16 | 1.92 | 5.71E-02 |
| age | 0.045 | 0.52 | 6.03E-01 |
| bmi | 0.15 | 1.75 | 8.19E-02 |
| bsa_dd | 0.2 | 2.34 | 2.09E-02 |
| bsa_m | 0.2 | 2.36 | 1.95E-02 |
| ckdepi2021 | 0.44 | 5.74 | 5.79E-08 |
| ckdepi2021mlmin_bsa_dd | 0.59 | 8.54 | 2.26E-14 |
| ckdepi2021mlmin_bsa_m | 0.59 | 8.62 | 1.48E-14 |
| clcr_adjbw | 0.42 | 5.43 | 2.49E-07 |
| clcr_dw | 0.5 | 6.84 | 2.38E-10 |
| clcr_ibw | 0.31 | 3.87 | 1.68E-04 |
| clcr_lbw | 0.51 | 6.91 | 1.67E-10 |
| clcr_wt | 0.49 | 6.57 | 9.53E-10 |
| dw | 0.17 | 2.07 | 4.07E-02 |
| ekfc | 0.46 | 6.11 | 9.83E-09 |
| ekfc_mlmin_bsa_dd | 0.59 | 8.47 | 3.31E-14 |
| ekfc_mlmin_bsa_m | 0.59 | 8.52 | 2.60E-14 |
| ht | 0.078 | 0.91 | 3.63E-01 |
| ibw | 0.078 | 0.92 | 3.62E-01 |
| lbw | 0.15 | 1.82 | 7.15E-02 |
| scr | -0.48 | -6.45 | 1.74E-09 |
| wt | 0.18 | 2.17 | 3.18E-02 |

**Table S5.** Comparison of different kidney function equations as covariates of CL with the best identified structural model based on the greatest reduction in the Akaike Information Criterion (AIC) value and confidence in parameter estimates

| Model Number | Structure, Error Model (distribution) | Estimated Parameters | AIC value | Δ AIC  (relative to Base) | Δ AIC  (relative to Model 2) |
| --- | --- | --- | --- | --- | --- |
| Base model | 1-compartment, Combined1 (normal) Fixed F, V1 and V2, | CL_TOL_ CL_TAZ_ | 4521.41 | 0 |  |
| Base model | Proposal: Combined2 error model, CL_TOL_ CL_TAZ_ correlated & ckdepi2021_bsa_dd as covariate of both CL_TOL_ CL_TAZ_ | | | |  |
| 1 | 1-compartment, Combined2 (normal) Fixed F, V1 and V2, CL_TOL_-CL_TAZ_ correlated | CL_TOL_-β_ckdepi2021^a^  CL_TAZ_-β_ckdepi2021^a^ | 4287.38 | -234.03 | 24.45 |
| **2** | **1-compartment, Combined2 (normal) Fixed F, V1 and V2, CL_TOL_-CL_TAZ_ correlated** | **CL_TOL_ -β_ckdepi2021_bsa_dd^a^**  **CL_TAZ_ -β_ckdepi2021_bsa_dd^a^** | **4262.93** | **-258.48** | **0** |
| 3 | 1-compartment, Combined2 (normal) Fixed F, V1 and V2, CL_TOL_-CL_TAZ_ correlated | CL_TOL_-β_ckdepi2021_bsa_m^a^  CL_TAZ_-β_ckdepi2021_bsa_m^a^ | 4263.69 | -257.72 | 0.76 |
| 4 | 1-compartment, Combined2 (normal) Fixed F, V1 and V2, CL_TOL_-CL_TAZ_ correlated | CL_TOL_-β_clcr_wt^a^  CL_TAZ_-β_clcr_wt^a^ | 4263.45 | -257.96 | 0.52 |
| **5** | **1-compartment, Combined2 (normal) Fixed F, V1 and V2, CL_TOL_-CL_TAZ_ correlated** | **CL_TOL_-β_clcr_lbw^a^**  **CL_TAZ_-β_clcr_lbw^a^** | **4261.64** | **-259.77** | **-1.29** |
| 6 | 1-compartment, Combined2 (normal) Fixed F, V1 and V2, CL_TOL_-CL_TAZ_ correlated | CL_TOL_-β_clcr_ibw^c^ | 4289.76 | -231.65 | 26.83 |
| 7 | 1-compartment, Combined2 (normal) Fixed F, V1 and V2, CL_TOL_-CL_TAZ_ correlated | CL_TOL_-β_clcr_adjbw^c^ | 4282.24 | -239.17 | 19.31 |
| **8** | **1-compartment, Combined2 (normal) Fixed V1, CL_TOL_-CL_TAZ_ correlated** | **CL_TOL_-β_clcr_dw^c^** | **4262.46** | **-258.95** | **-0.47** |
| 9 | 1-compartment, Combined2 (normal) Fixed V1, CL_TOL_-CL_TAZ_ correlated | CL_TOL_-β_EKFC^c^ | 4285.83 | -235.58 | 22.9 |
| 10 | 1-compartment, Combined2 (normal) Fixed V1, CL_TOL_-CL_TAZ_ correlated | CL_TOL_-β_EKFC_bsa_dd^c^ | 4264.21 | -257.2 | 1.28 |
| **11** | **1-compartment, Combined2 (normal) Fixed V1, CL_TOL_-CL_TAZ_ correlated** | **CL_TOL_-β_EKFC_bsa_m^c^** | **4261.36** | **-260.05** | **-1.57** |

^a^(eGFR/60), models in bold denoting lowest AIC and all within 2 points of Model 2

**Table S6.** Parameter estimates of the final pharmacokinetic model

|  | **VALUE** | | **STOCH. APPROX.** | | | |
| --- | --- | --- | --- | --- | --- | --- |
|  |  |  | **S.E.** | **R.S.E.(%)** | **P2.5** | **P97.5** |
| **Fixed Effects** | | | | | | |
| V1_pop | 32.1 | |  |  |  |  |
| Cltol_pop | 2.78 | | 0.16 | 5.74 | 2.48 | 3.11 |
| beta_Cltol_logtCkdepi2021mlmin_bsa_dd | 0.74 | | 0.081 | 10.9 | 0.58 | 0.9 |
| Cltaz_pop | 6.73 | | 0.45 | 6.68 | 5.91 | 7.67 |
| beta_Cltaz_logtCkdepi2021mlmin_bsa_dd | 0.84 | | 0.093 | 11.1 | 0.66 | 1.03 |
| V2_pop | 43.3 | |  |  |  |  |
| F_pop | 0.33 | |  |  |  |  |
| Standard Deviation of the Random Effects | | | | | | |
|  | Value | C.V.(%) |  |  |  |  |
| omega_V1 | 0.42 | 43.92 |  |  |  |  |
| omega_Cltol | 0.61 | 66.76 | 0.044 | 7.28 | 0.53 | 0.7 |
| omega_Cltaz | 0.69 | 77.98 | 0.055 | 7.98 | 0.59 | 0.81 |
| omega_V2 | 0.38 | 39.41 |  |  |  |  |
| **Correlations** | | | | | | |
| corr_Cltol_Cltaz | 0.88 | | 0.035 | 3.96 | 0.79 | 0.93 |
| **Error Model Parameters** | | | | | | |
| a1 | 8.07 | | 0.94 | 11.7 | 6.44 | 10.12 |
| b1 | 0.23 | | 0.025 | 10.7 | 0.19 | 0.28 |
| a2 | 1.3 | | 0.2 | 15.4 | 0.97 | 1.75 |
| b2 | 0.41 | | 0.029 | 6.89 | 0.36 | 0.47 |

**Table S7.** Distribution of participants across estimated glomerular filtration rate groups including central tendency and variance of the population to support simulations

| eGFR Group | N | Population% | Mean (mL/min) | Standard Deviation | Coefficient of Variation |
| --- | --- | --- | --- | --- | --- |
| <15 mL/min | 7 | 5% | 10 | 4 | 36% |
| 15-29 mL/min | 18 | 13% | 26 | 3 | 12% |
| 30-50 mL/min | 17 | 12% | 42 | 5 | 11% |
| 51-120 mL/min | 79 | 57% | 90 | 19 | 21% |
| >120 mL/min | 18 | 13% | 138 | 18 | 13% |

**Figure S2**. Ceftolozane (A) and Tazobactam (B) population-predictions vs. observations and individual-predictions vs. observations for the final population pharmacokinetic model


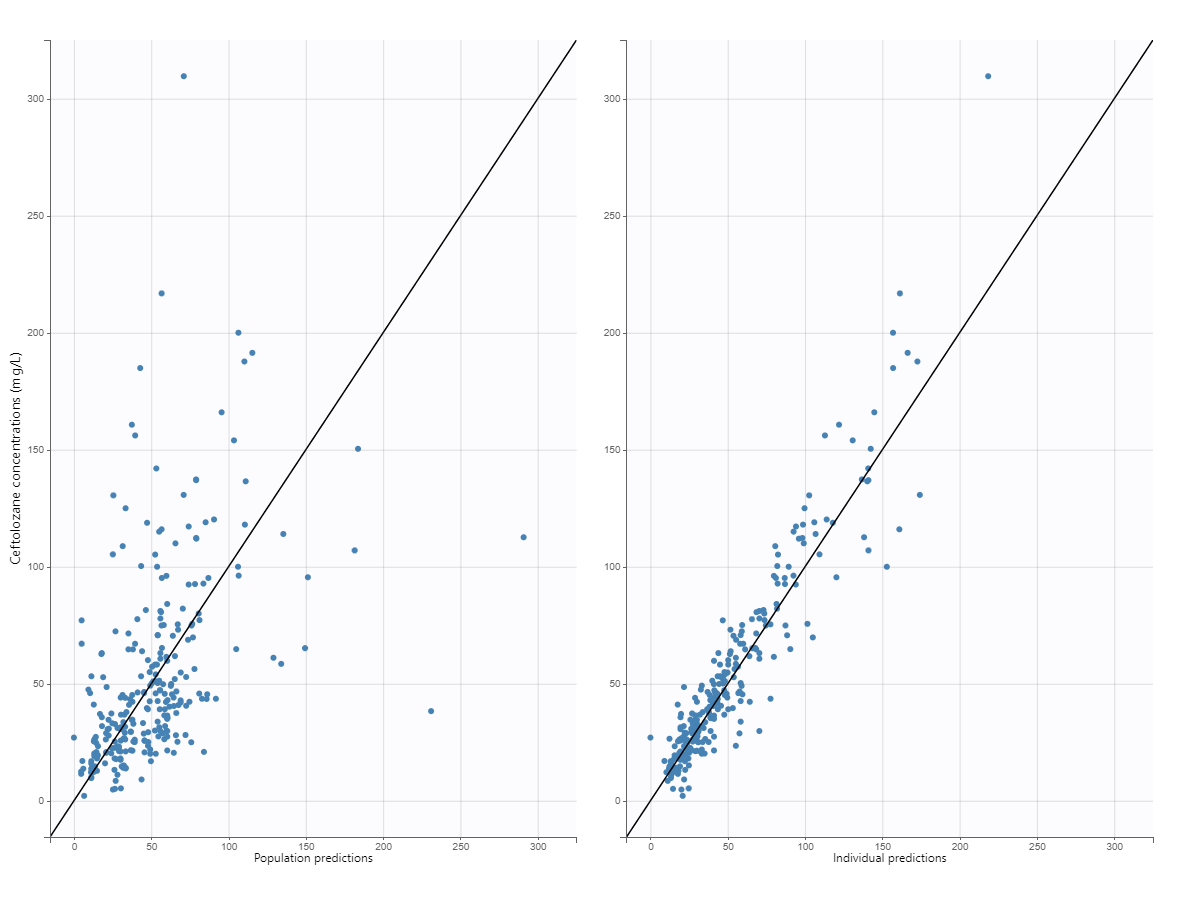
A


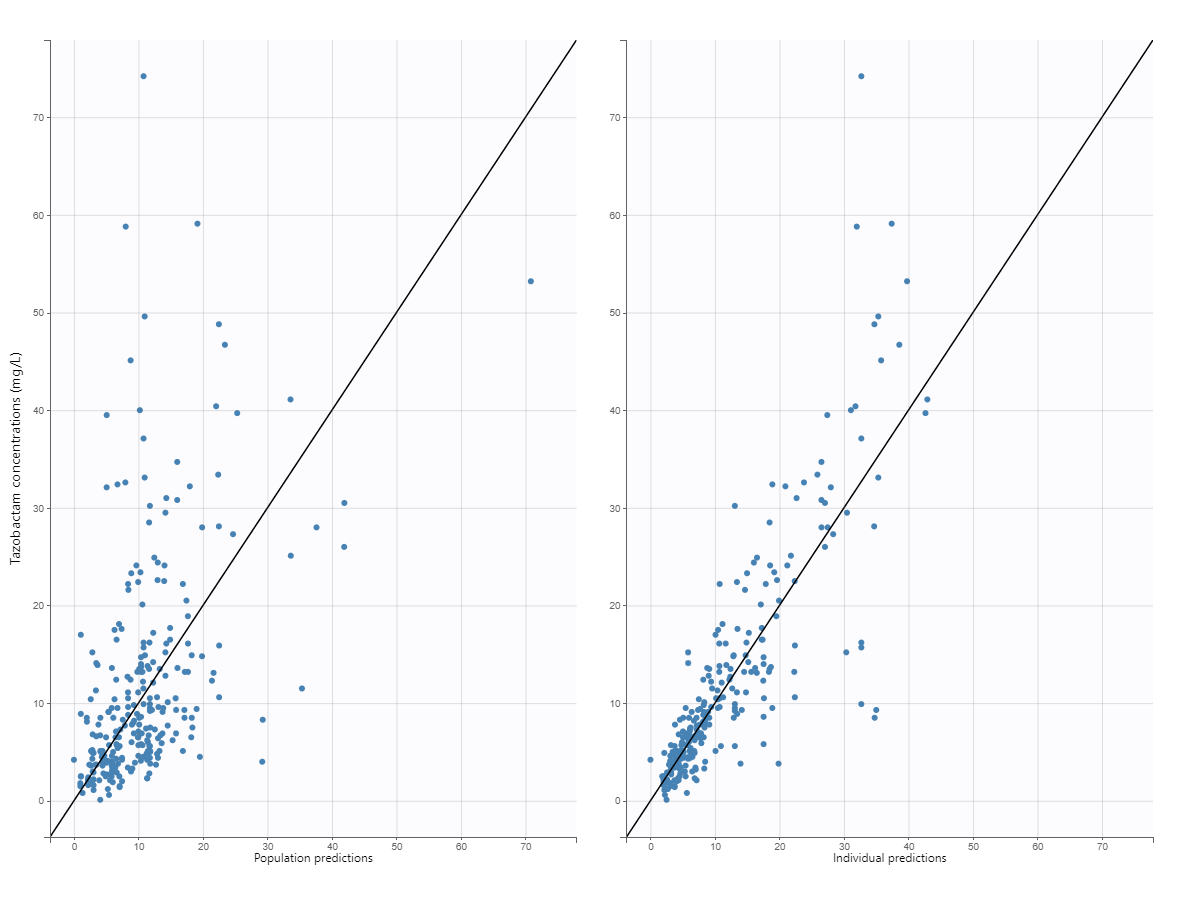
B

**Figure S3**. Scatter plot of the residual vs. time and of the residual vs. individual predictions of ceftolozane (A) and tazobactam (B) for the final population pharmacokinetic model


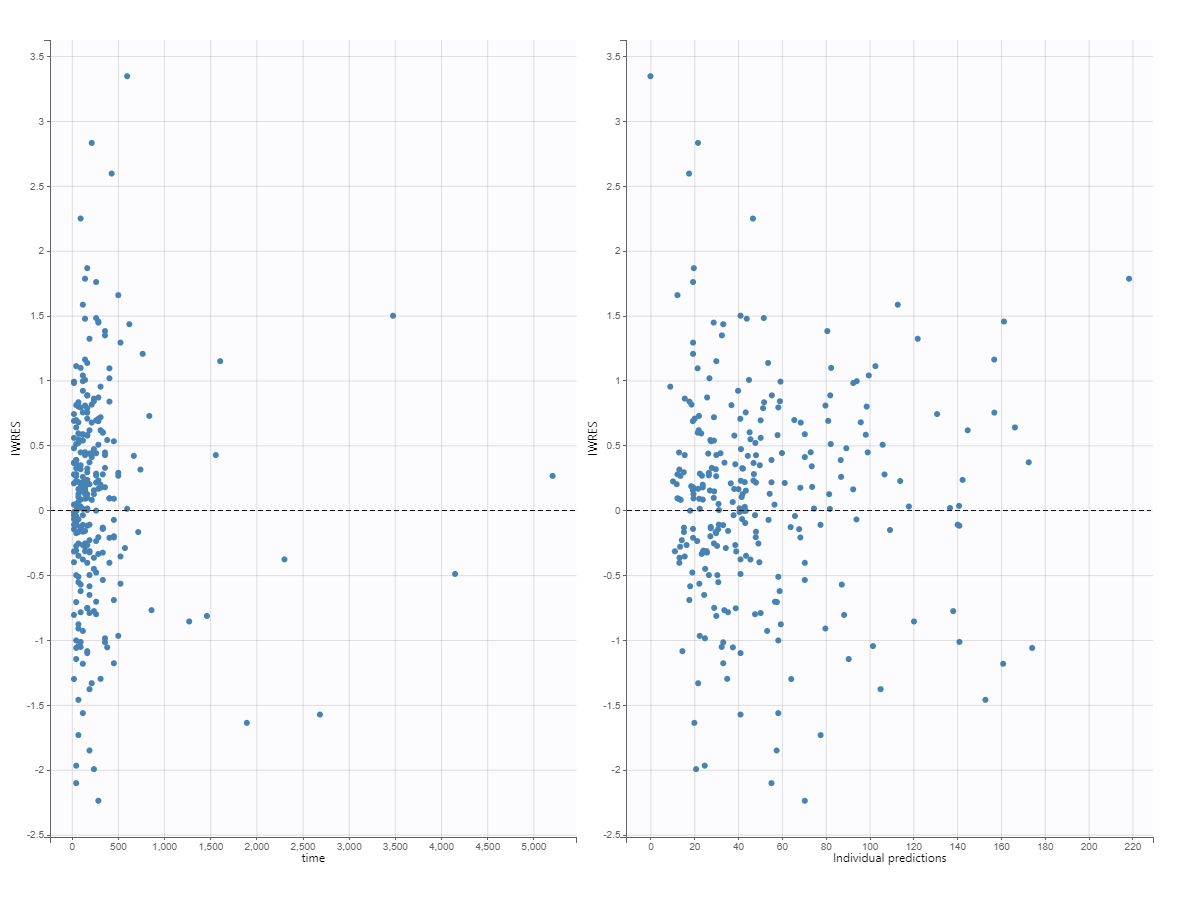
A
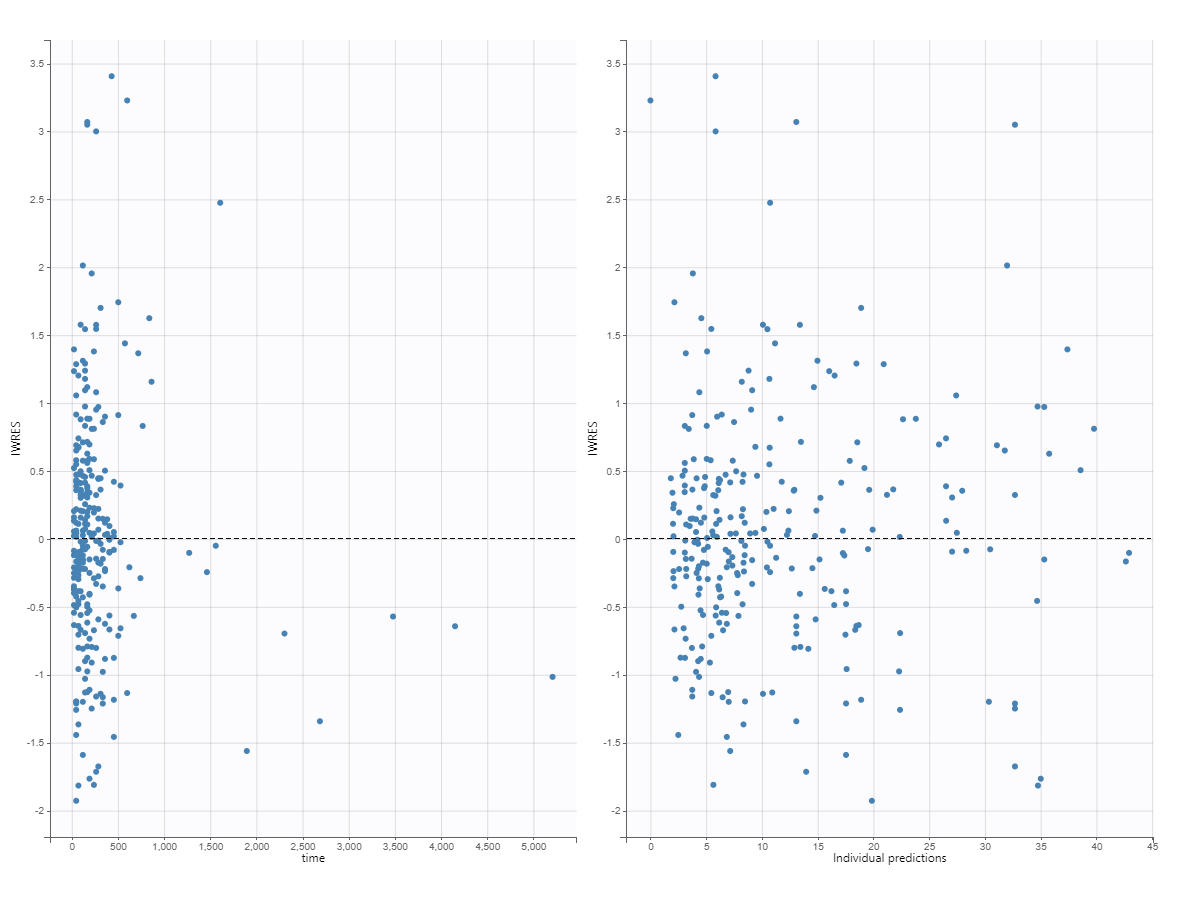
B

**Figure S4**. Distribution of the Individual Weighted Residuals and of the Normalized Prediction Distribution Errors of ceftolozane (A) and tazobactam (B) for the final population pharmacokinetic model


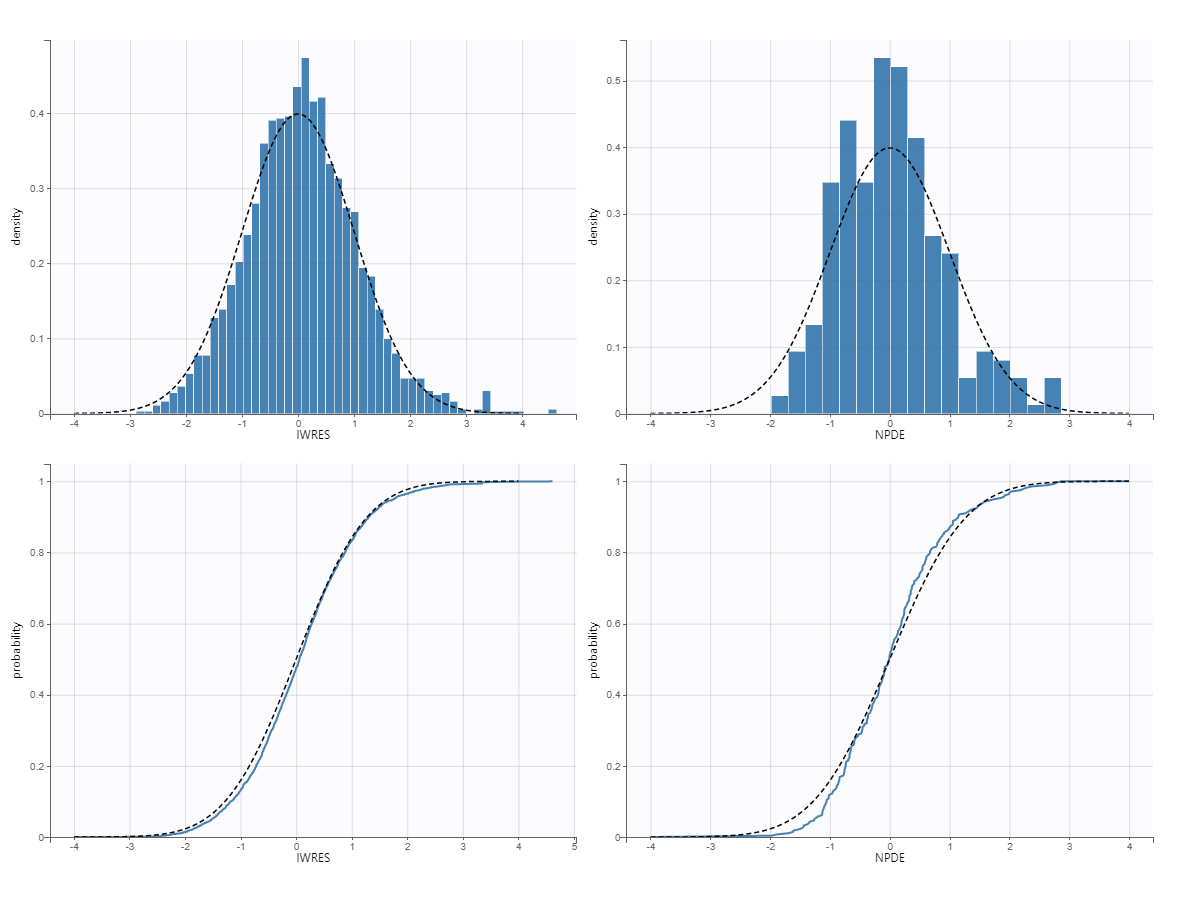
A
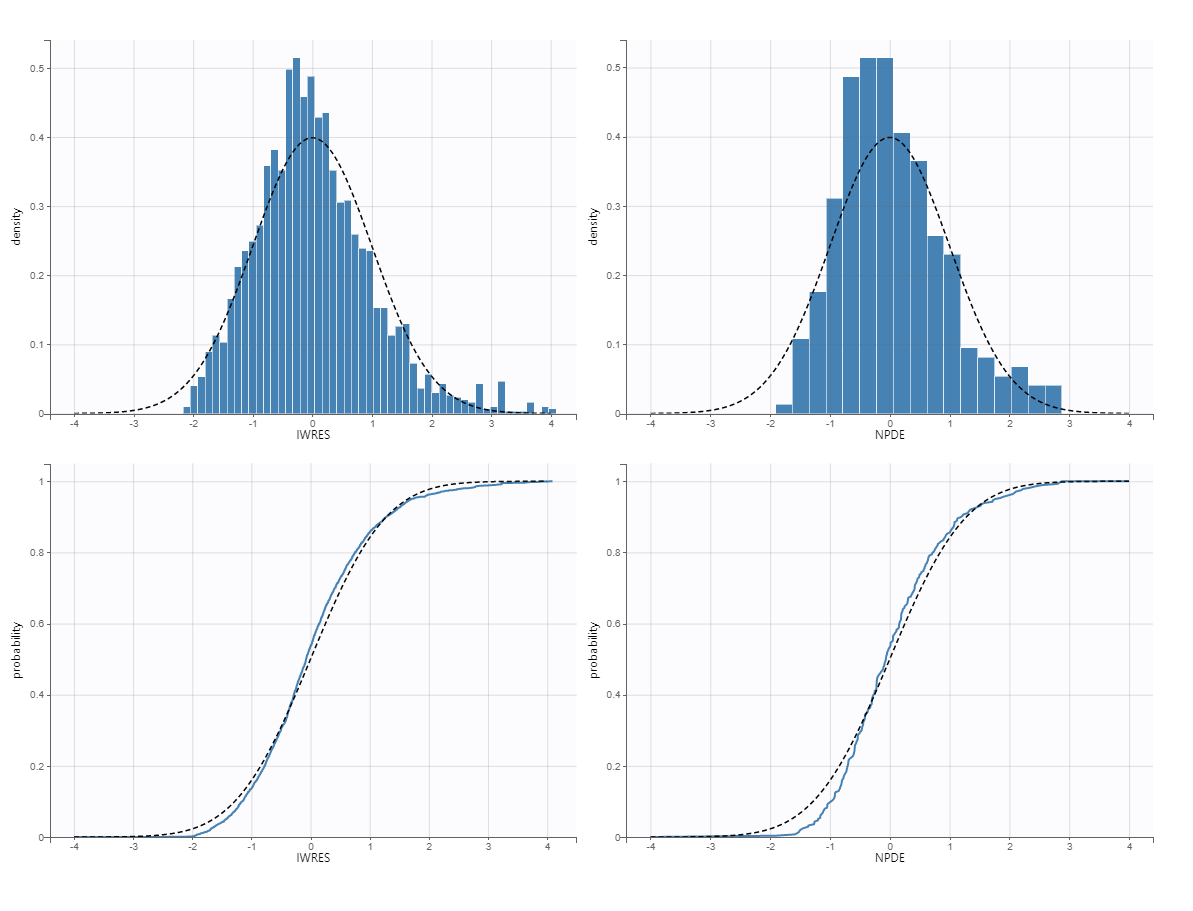
B

**Figure S5**. Visual predictive check (VPC) for the ceftolozane (A) and tazobactam(B) final population pharmacokinetic model


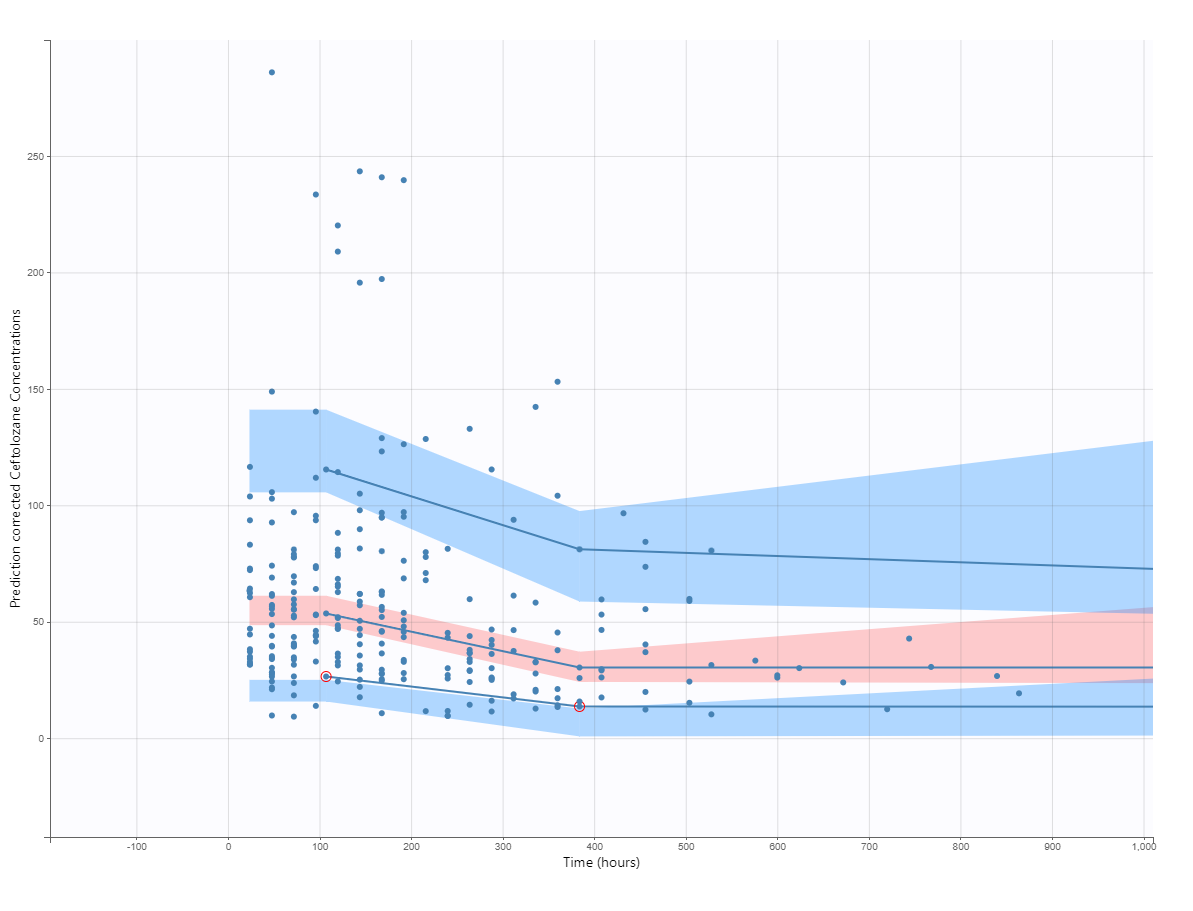
A
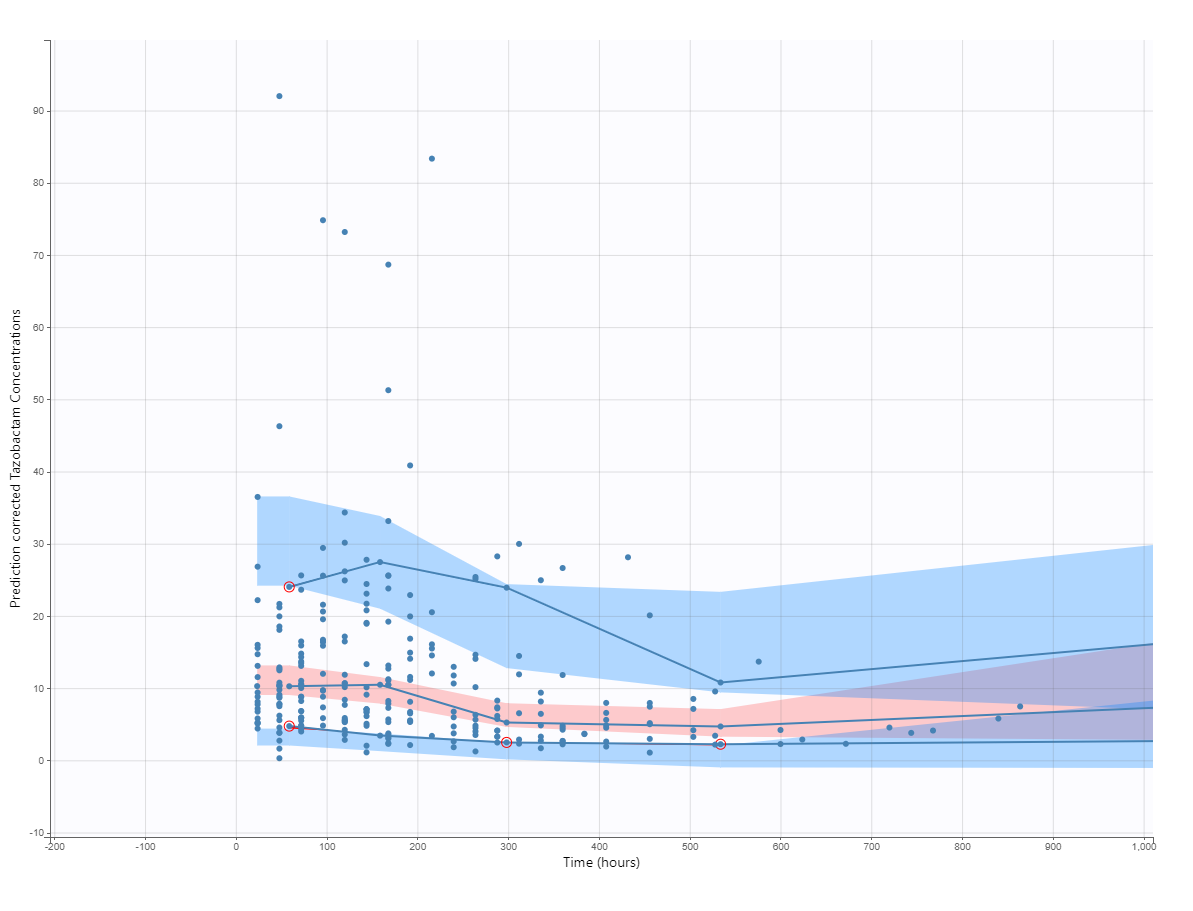
B

**Figure S6**. Distribution of the individual parameters


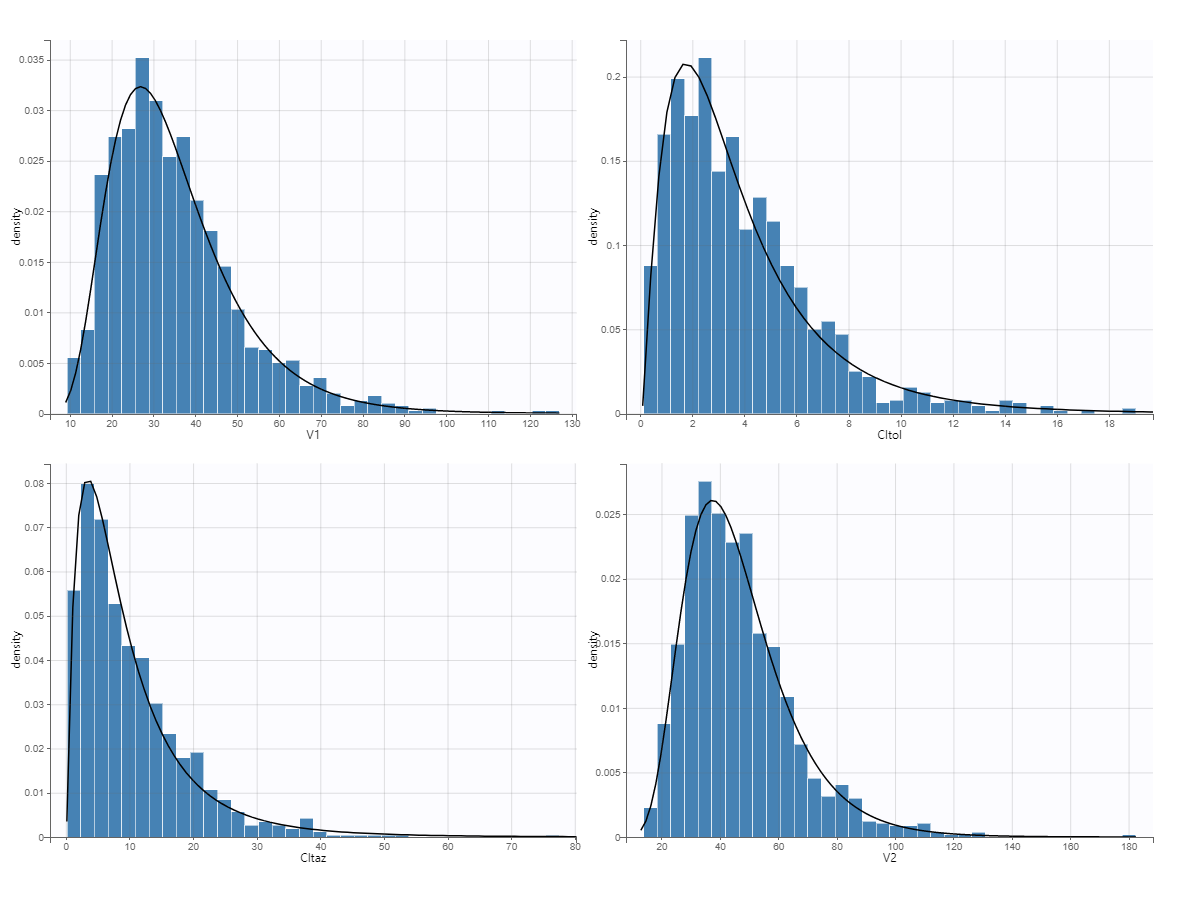

Supplement: Supplemental material — Tables S1 to S7; Fig. S1 to S6. [file aac.01215-25-s0001.docx]
